# A Comparison of Positron Emission Tomography and Colonoscopy for the Detection of Advanced Colorectal Neoplasms in Subjects Undergoing a Health Check-Up

**DOI:** 10.1371/journal.pone.0069111

**Published:** 2013-07-19

**Authors:** Shu-Wei Huang, Chen-Ming Hsu, Wen-Juei Jeng, Tzu-Chen Yen, Ming-Yao Su, Cheng-Tang Chiu

**Affiliations:** 1 Department of Gastroenterology and Hepatology, Chang Gung Memorial Hospital at Linkou, Taoyuan, Taiwan; 2 Department of Nuclear Medicine, Chang Gung Memorial Hospital at Linkou and Chang Gung University College of Medicine, Taoyuan, Taiwan; Wayne State University, United States of America

## Abstract

**Background & Aims:**

There is no agreement as to whether F-18 fluorodeoxyglucose positron emission tomography and computed tomography (FDG PET/CT) screening for advanced colorectal neoplasms is meaningful. This retrospective study was undertaken to determine whether FDG PET/CT may be a valuable screening tool for the detection of advanced colorectal neoplasms.

**Methods:**

A retrospective review of the records of 1,109 FDG PET/CT scans acquired from January 2007 to December 2011 was performed. Colonoscopy and FDG PET/CT imaging were performed within two days of each other. The results of colonoscopy were taken as the gold standard, either with or without the results of the histopathological examination. An advanced neoplasm was defined as the presence of a malignant tumor, an adenoma ≥1 cm, or histological evidence of high-grade dysplasia or significant villous components.

**Results:**

A total of 36 subjects had advanced colorectal neoplasms detected by colonoscopy (totaling 38 neoplasms). Six of the 38 neoplasms were also detected by FDG PET/CT. The sensitivity, specificity, positive predictive value, negative predictive value, and overall accuracy of FDG PET/CT in the detection of advanced colorectal neoplasms were 15.8% (6/38), 99.1% (1063/1073), 37.5% (6/16), 97.1% (1063/1095), and 96.2% (1069/1111) respectively. The presence of lesions with an endoscopic size ≤1.5 cm (P<0.001) and low-grade dysplasia (P<0.001) were the main predictors of false-negative FDG PET/CT findings.

**Conclusions:**

We conclude that FDG PET/CT screening of advanced colorectal neoplasms is unwarranted, especially in the presence of lesions with an endoscopic size ≤1.5 cm or low-grade dysplasia.

## Introduction

Colorectal cancer (CRC) is the third most common malignant neoplasm worldwide and one of the most frequent cause of cancer-related death [1]. Over the years, there has been growing interest in screening as a means for reducing CRC-related mortality in average-risk asymptomatic individuals [2], [3]. Advanced colorectal neoplasms include advanced adenomas (adenomas ≥1 cm or containing high-grade dysplasia or significant villous component) and malignant tumors, which are the main target lesions of CRC screening [2]. Advanced adenomas represent high-risk precancerous lesions [3]. According to the traditional adenoma-carcinoma sequence, the majority of CRCs arise from benign adenomas. The dwell time of a benign adenoma to its transformation into a CRC has been projected to be approximately 5–10 years [4].

For clinicians and researchers a key consideration is which means is the most appropriate for CRC screening in asymptomatic individuals. Conventional colonoscopy is the current screening standard for the detection of precancerous adenomas and CRCs [5]. Previous studies have shown that the detection and removal of premalignant adenomas may prevent incident CRCs and could substantially reduce CRC mortality [6], [7]. Unfortunately, colonoscopy is an invasive procedure which requires an uncomfortable bowel preparation and carries potential risks of complications (perforation, cardiopulmonary events) [3].

Fluorine-18-2-ﬂuoro-2-deoxy-D-glucose positron emission tomography and computed tomography (FDG PET/CT) is a noninvasive, painless molecular imaging technology which can noninvasively survey the entire body and sensitively detect numerous cancers. Although previous studies have shown the utility of this technique for initial staging and restaging of CRC patients [8], there is no agreement as to whether FDG PET/CT screening for advanced colorectal neoplasms is meaningful. This retrospective study was undertaken to determine whether FDG PET/CT may be a valuable screening tool for the detection of advanced colorectal neoplasms. We also sought to identify the potential sources of false-negative FDG PET/CT findings.

## Patients and Methods

### Study Design and Participants

A retrospective review of the records of FDG PET/CT scans acquired from January 2007 to December 2011 was performed. All of the study participants were consecutively enrolled among subjects undergoing a health check-up offered by the Chang Gung Healthcare Center. The Center proposed a variety of examinations that were chosen by the participant him/herself. It was a self-paid check-up that was not part of a routine screening. To be included in the study, subjects were required to have colonoscopy and FDG PET/CT imaging performed within two days of each other. Subjects with fasting glucose levels >200 mg/dL, a positive history of previous colon resections, or who failed to achieve cecal intubation during colonoscopy were excluded. The inclusion and exclusion criteria were met by a total of 1,109 subjects. Age, sex, serum glucose levels, weight, and height were recorded. The body mass index (BMI) was calculated by dividing the body weight (in kilograms) by the height (in meters) squared.

The Institutional Review Board waived the need for informed consent from the participants because this research was a retrospectively observational analysis, and the identifying information was not included in the collected data. This study was approved by the Institutional Review Board of the Chang Gung Memorial Hospital.

### FDG PET/CT Imaging

All of the FDG PET/CT scans were performed using a combined PET/CT scanner (Discovery ST16; GE Health Systems, Milwaukee, WI, USA). The participants were instructed to fast for at least 6 hours before examination. The scan started approximately 50 min after the injection of 370±10% MBq of ^18^F-FDG. A diluted CT contrast agent (iothalamate meglumine; Mallinckrodt Inc, Hazelwood, MO, USA) was administered orally during the tracer uptake period in the presence of FDG-avid lesions located in the gastrointestinal tract. Participants were examined in the supine position. All of the participants received a non-enhanced CT scan from the head to the proximal thigh. Immediately after the CT acquisition, the PET emission scans were acquired in the two-dimensional mode. PET images were reconstructed using CT attenuation maps. The standardized uptake value (SUV) was defined as the tissue concentration (MBq/mL) of the tracer divided by the activity injected per body weight (MBq/g). The maximum SUV in the volume of interest was considered as the SUVmax for the purpose of analysis. On the day following the scans, the images were interpreted by experienced nuclear medicine physicians who were unaware of the colonoscopy results.

### Colonoscopy

Within two days of FDG PET/CT imaging, the study participants underwent colonoscopy (CF260L, Olympus, Tokyo, Japan) under deep sedation. All of the participants received 4 L/min of supplemental oxygen delivered through a nasal cannula. Physiological monitoring included pulse oximetry, electrocardiography, heart rate, and automated blood pressure measurements. The administration of sedative agents (propofol alone or propofol in combination with fentanyl or alfentanil, and/or midazolam) was performed by staff anesthesiologists. The study participants were instructed to take a colon preparation agent (either 2 L of polyethylene glycol (PEG) electrolyte solution or split-dose aqueous sodium phosphate solution) the day before the examination. The use of PEG for colon preparation was recommended when sodium phosphate was contraindicated. The colonoscopy records included the quality of bowel preparation, location, size, and type of the polyps. The quality of the bowel preparation was graded by the endoscopists as: (1) excellent, absence or near absence of fecal material in the colon and/or small amounts of ﬂuid; (2) good, small amounts of thin fecal material seen and suctioned easily; (3) fair, moderate amounts thick liquid to semisolid fecal material seen and suctioned, >90% of mucosa seen; (4) poor, large amounts of solid fecal material found, <90% of mucosa seen [9], [10]. The lesion size was determined by comparison to the size of an opened endoscopic forceps. According to their appearance, polyps were classified as pedunculated (type 0-Ip) or non-pedunculated (sessile, 0-Is; slightly elevated, 0-IIa; flat, 0-IIb; slightly depressed, 0-IIc; excavated, 0-III) [11]. Malignant lesions were excluded from this morphological classification. The vascular network of the polyps was examined using a narrow band imaging (NBI) system (Olympus, Tokyo, Japan). Polyps with a brownish vascular network enhanced by NBI were considered as adenomatous lesions [12]. After obtaining the participant’s consent, the polyps were removed by polypectomy or underwent biopsy for the histolopathological examination. All of the specimens were examined by experienced pathologists to interpret the histological type and the degree of dysplasia [13], [14]. An advanced colorectal neoplasm was defined as the presence of a malignant tumor, an adenoma ≥1 cm, or histological evidence of high-grade dysplasia or significant villous components [2], [3]. When a CRC was diagnosed, the patient was referred for surgery.

### Classification of FDG PET Findings

FDG PET/CT findings were considered as true-positive in the presence of focal hypermetabolic lesions in subjects with evidence of advanced neoplasms in a compatible location on colonoscopy. FDG PET/CT findings were considered as false-positive in the presence of focal hypermetabolic lesions in subjects without evidence of advanced neoplasms in a compatible location on colonoscopy. The results of FDG PET/CT were considered as false-negative in subjects who did not show focal hypermetabolic lesion but who had evidence of advanced neoplasms on colonoscopy. The results of FDG PET/CT were considered as true-negative in subjects who did not show focal hypermetabolic lesions and in the absence of advanced neoplasms on colonoscopy.

### Statistical Analysis

Quantitative data are presented as means and standard deviations or medians, whereas categorical variables are expressed as rates and proportions. We calculated the sensitivity, specificity, positive predictive value, negative predictive value, and overall accuracy of FDG PET/CT for the detection of colorectal neoplasms with the corresponding 95% confidence intervals. The χ^2^ test was used to identify the factors associated with the detection of advanced neoplasms on FDG PET/CT scans. The Fisher’s exact test was used when the expected count was less than five. We used the Mann-Whitney *U* test to compare SUVmax values (subjects with true-positive *vs.* those with false-positive FDG PET/CT findings; subjects with adenocarcinomas *vs.* those with non-malignant advanced adenomas). Two-tailed *P* values <0.05 were considered statistically significant.

## Results

Table 1 shows the general characteristics of the study subjects. The proportion of excellent, good, fair and poor bowel preparation were 3.5% (39/1109), 41.7% (462/1109), 44.3% (491/1109), and 10.5% (117/1109) respectively. Among the 1,109 participants, colonoscopy detected a total of 284 non-advanced adenomatous polyps in 193 men and 91 women. Moreover, a total of 38 advanced neoplasms (including 5 adenocarcinomas) were identified by colonoscopy in 26 men and 10 women. Two of the 26 men had more than one advanced neoplasm. The adenoma detection rate (ADR) was 28.5% in the entire study population (33.1% in males and 21.9% in females, *P*<0.001). The prevalence rates of advanced neoplasms and CRCs were 3.4% and 0.45%, respectively. Among the 317 adenomas, 10 (3.2%) were diagnosed by endoscopy with NBI instead of the pathological results (including 4 polyps ≥ 1 cm in size which were considered as advanced neoplasms).

**Table 1:** General characteristics of the study participants (n  = 1109).

Parameters	
***Sex***	
Males	661 (59.6%)
Females	448 (40.4%)
***Age (years)***	
Mean (range)	53.2±10.3 (20–81)
Males	53.1±10.2
Females	53.3±10.4
***Body mass index, kg/m^2^***	
Mean (range)	24.5±3.4 (15.5–38.0)
Males	25.0±3.4
Females	23.6±3.2
<23	369 (33.3%)
23–25	297 (26.8%)
≥25	443 (39.9%)
***Fasting serum glucose level (mg/dL)***	95.8±16.4
<100	842 (75.9%)
100–125	202 (18.2%)
126–200	65 (5.9%)

FDG PET/CT yielded 16 positive results in this study, including 6 true-positive and 10 false-positive findings. A total of 32 advanced neoplasms detected by colonoscopy were not identified on FDG PET/CT scans (false-negative findings). The lesions missed on FDG PET/CT included one polypoid cancer (1.5 cm in size) located in the sigmoid-colon and another semi-annularly ulcerative cancer (3 cm in size) located in the rectum. The sensitivity, specificity, positive predictive value, negative predictive value and overall accuracy of FDG PET/CT for the detection of advanced neoplasms were 15.8% (6/38), 99.1% (1063/1073), 37.5% (6/16), 97.1% (1063/1095), and 96.2% (1069/1111), respectively. The reliability and the predictive values of FDG PET/CT for the identification of different colorectal neoplasms are presented in Table 2.

**Table 2:** Sensitivity, specificity, positive predictive value, negative predictive value, and overall accuracy of FDG PET/CT for the detection of different colorectal neoplasms.

Type of colorectalneoplasm	Sensitivity,%(95% CI^a^)	Specificity,% (95% CI)	PPV^b^,% (95% CI)	NPV^c^,% (95% CI)	Accuracy,% (95% CI)
All neoplasms	2.5 (0.8–4.2) [8/322]	99.0 (98.3–99.7) [781/789]	50.0 (25.5–74.5) [8/16]	71.3 (68.7–74.0) [781/1095]	71.0 (68.4–73.7) [789/1111]
Non-advanced neoplasms	0.7 (−0.3–1.7) [2/284]	99.0 (98.3–99.7) [781/789]	20.0 (−4.8–44.8) [2/10]	73.5 (70.8–76.1) [781/1063]	73.0 (70.3–75.6) [783/1073]
Advanced neoplasms	15.8 (4.2–27.4) [6/38]	99.1 (98.5–99.6) [1063/1073]	37.5 (13.8–61.2)[6/16]	97.1 (96.1–98.1) [1063/1095]	96.2 (95.1–97.3) [1069/1111]
≥1.0 cm in size^d^	20.7 (6.0–35.4) [6/29]	99.1 (98.5–99.6) [1063/1073]	37.5 (13.8–61.2)[6/16]	97.9 (97.0–98.7) [1063/1086]	97.0 (96.0–98.0) [1069/1102]
>1.5 cm in size^e^	71.4 (38.0–104.9) [5/7]	99.1 (98.5–99.6) [1063/1073]	33.3 (9.5–57.2) [5/15]	99.8 (99.6–100.1)[1063/1065]	98.9 (98.3–99.5) [1068/1080]
Adenocarcinomas^f^	60.0 (17.1–102.9) [3/5]	99.1 (98.5–99.6) [1063/1073]	23.1 (0.2–46.0) [3/13]	99.8 (99.6–100.0)[1063/1065]	98.9 (98.3–99.5) [1066/1078]

a CI, confidence interval.

b PPV, positive predictive value.

c NPV, negative predictive value.

d Advanced neoplasms <1.0 cm were excluded from this analysis.

e Advanced neoplasms ≤1.5 cm were excluded from this analysis.

f Non-malignant advanced neoplasms were excluded from this analysis.

**Figure 1:**
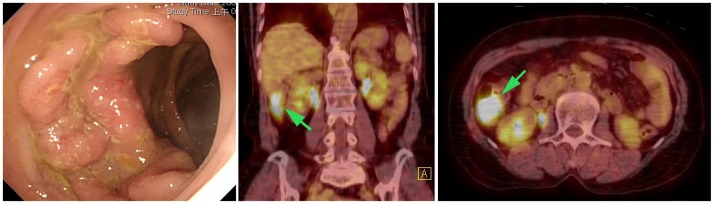
One ulcerated cancer with well-demarcated borders (4.0 cm in size) was identified by colonoscopy in the ascending colon. The results of colonoscopy were taken as the gold standard. FDG PET/CT imaging revealed an increased FDG uptake in a compatible location (arrow); therefore, the FDG PET/CT findings were considered as true-positive.

**Figure 2:**
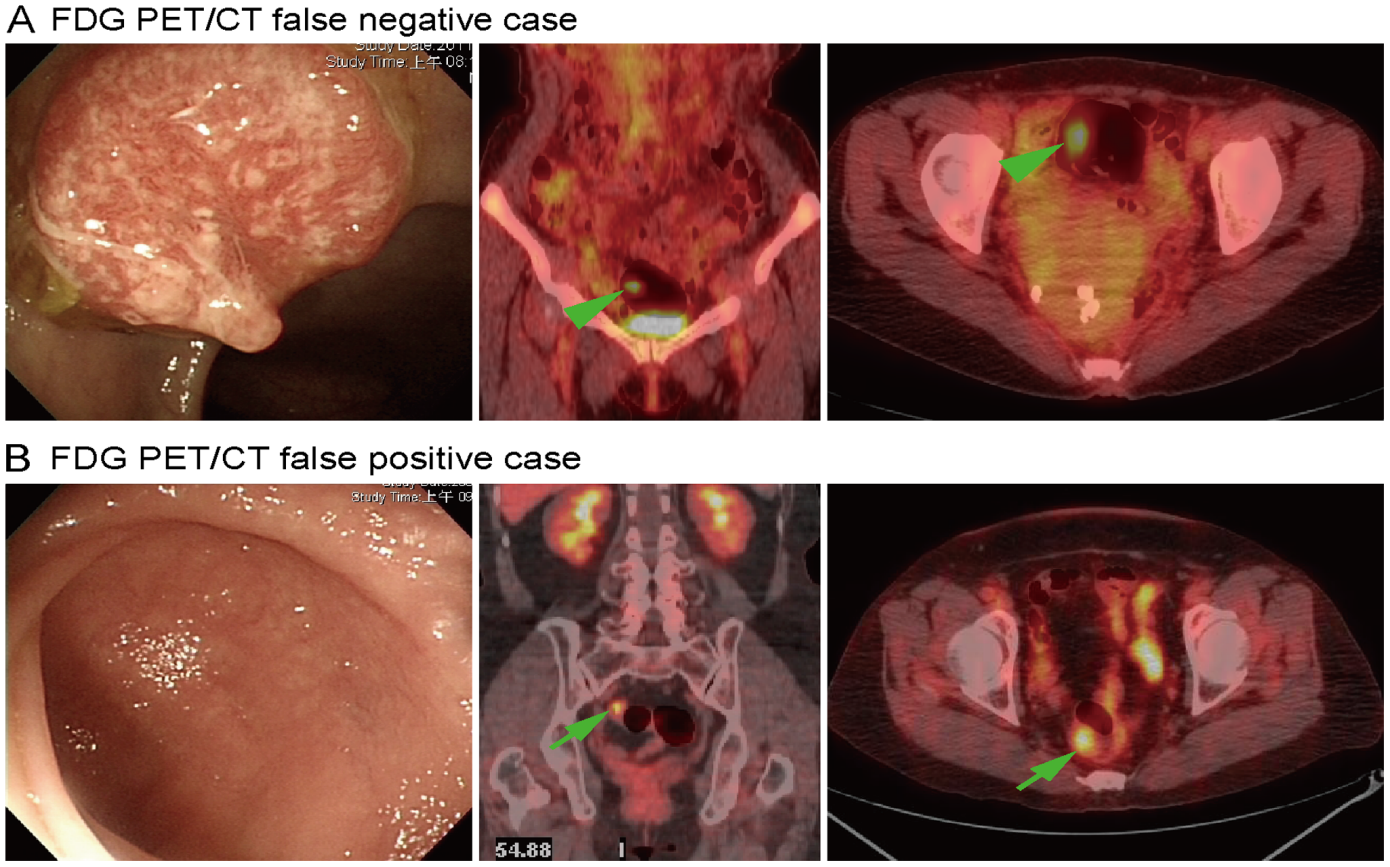
Representative cases of false-negative (panel A) and false-positive (panel B) FDG PET/CT findings. (A) One polypoid cancer (1.5 cm in size) was identified by colonoscopy in the sigmoid colon. FDG PET/CT scans revealed an increased FDG uptake in the luminal air (arrowhead). This result was erroneously interpreted as a negative finding because of misregistration. (B) Colonoscopy revealed a normal mucosa. However, FDG PET/CT showed an increased FDG uptake in the sigmoid colon (arrow), which was erroneously interpreted as a positive result.

The factors unfavorably associated with the likelihood of true-positive FDG PET/CT findings included tumor size (≤1.5 cm *vs.* >1.5 cm, 3.3% *vs.* 71.4%, *P*<0.001) and the degree of dysplasia (low grade *vs.* high grade/adenocarcinoma, 0% *vs.* 71.4%, *P*<0.001) (Table 3). The median SUVmax values of the participants with true-positive and false-positive FDG PET/CT findings were 8.6 and 5.6, respectively (*P* = 0.057). The median SUVmax values of subjects with adenocarcinomas and non-malignant advanced adenomas were 25.0 and 6.7, respectively (*P* = 0.05). The overall rate of positive FDG PET/CT results was 1.4% (16/1111). Eleven of the 16 subjects (68.8%) who had positive FDG PET/CT results also showed positive findings on colonoscopy. Figure 1 shows a representative case with true-positive FDG PET/CT results, whereas Figure 2 shows two representative cases with false-negative (panel A) and false-positive (panel B) FDG PET/CT findings. The detailed characteristics of subjects with true-positive, false-positive, and false-negative FDG PET/CT findings are reported in Table S1.

**Table 3:** Sensitivity of FDG-PET for the detection of advanced colorectal neoplasms according to different clinicopathological characteristics (n  = 38).

Parameters	Sensitivity (%)	True-positive FDG-PETfindings (n = 6)	False-negative FDG-PET findings (n = 32)	*P*
***Endoscopic size***				<0.001
≤1.5 cm	3.3	1	30	
>1.5 cm	71.4	5	2	
***Endoscopic morphology*** a				0.40
Non-pedunculated	7.1	2	26	
Pedunculated	20.0	1	4	
***Degree of dysplasia*** b				<0.001
Low-grade dysplasia	0	0	27	
High-grade dysplasia/adenocarcinoma	71.4	5	2	
***Histological type*** a,b				0.51
Tubular	0	0	11	
Tubulovillous/villous	11.1	2^c^	16	
***Body mass index (kg/m^2^)***				0.36
<25	22.7	5	17	
≥25	6.3	1	15	
***Fasting serum glucose level (mg/dL)***				0.64
<100	19.2	5	21	
≥100	8.3	1	11	

a Adenocarcinomas were excluded.

b Pathological results were missing for four adenomas.

c Concomitant presence of high-grade dysplasia.

## Discussion

Although FDG PET/CT has proven value in the staging and restaging of CRC patients [8], [15], the question as to whether FDG PET/CT may be useful as a screening tool for advanced colorectal neoplasms remains open. Previous studies in the field have reported very wide detection rates, ranging from 20% to 90% [16]–[22]. Several factors may account for such discrepancies, including different inclusion criteria for FDG PET/CT imaging, the use of different screening protocols or gold standards, variable time intervals between FDG PET/CT scans and other screening tests, and the interobserver variability between different hospitals. Differently from previous studies, the study participants who attended a health check-up examination underwent both colonoscopy and FDG PET/CT within a very short time period (i.e., two days of each other). The prevalence rates of adenomas, advanced neoplasms, and CRCs were 28.5%, 3.4%, and 0.45%, respectively. Such rates are similar to those previously reported for average-risk populations [23], [24]. The overall prevalence of focal hypermetabolic lesions on FDG PET/CT was 1.4% in the entire study cohort (16/1111). Among these subjects, neoplastic lesions were observed in 50% (8/16) of the cases, whereas the prevalence of non-neoplastic lesions in the colon was 18.8% (3/16). These prevalence figures are in accordance with those previously reported in the literature [25]. Differently from previous studies which were focused on high-risk groups with advanced neoplasms or CRCs [18]–[20], we specifically investigated a general population sample. The inclusion of average-risk subjects may thus explain the lower detection rate of advanced neoplasms on FDG PET/CT scans observed in this report.

FDG PET/CT successfully identified only 6 of the 38 lesions detected by colonoscopy. More importantly, there were 10 false-positive and 32 false-negative FDG PET/CT scans. Several factors may influence the detection rate of advanced neoplasms by FDG PET/CT imaging. In this study, we found that an endoscopic size ≤1.5 cm and the presence of low-grade dysplasia were unfavorably associated with the likelihood of having positive FDG-PET/CT findings. These results are in accordance with those of two previous studies showing that the detection rates of FDG PET/CT are positively correlated with the size of advanced colorectal neoplasms and the degree of dysplasia [18], [19]. In general, the detection rates of FDG PET/CT imaging in the screening of colorectal lesions depend on size of lesions [18]–[20]. In the present study, we found that 30 of the 32 lesions missed on FDG PET/CT scans had a longest axis ≤1.5 cm according to the colonoscopy results. These findings are in keeping with those of Friedland *et al.* who showed that the sensitivity of FDG PET/CT was 22.9% for premalignant colon lesions with a longest axis <3 cm, and it was only 17% for colorectal cancers with a longest axis <2 cm [20]. A possible explanation for the lower sensitivity observed in our study and the report by Friendland et al. may be the different measurement procedures used during colonoscopy. It is noteworthy that only the longest axis of a lesion is measured upon colonoscopy detection. However, most of the lesions identified by colonoscopy screening have an irregular shape, with a shorter axis below the physical threshold for FDG PET/CT detection [26].

Another factor which was found to be significantly associated with a lower likelihood of positive FDG PET/CT findings was the presence of low-grade dysplasia. Because the large intestine is characterized by a physiological FDG uptake, a successful FDG PET/CT screening requires the occult lesions to have a higher uptake than the surrounding background. In this study, 27 of the 32 lesions associated with false-negative FDG PET/CT findings were characterized by the presence of low-grade dysplasia. Notably, the expression of glucose transporter I is markedly lower in low-grade than in high-grade neoplasms. This biochemical feature may result in a significantly lower uptake of FDG by lesions characterized by low-grade dysplasia, which could make them undistinguishable from the bowel background [27]. Previous studies have tried to identify other potential predictors of positive FDG PET/CT findings in a screening setting. In this regard, it has been shown that pedunculated lesions are more easily detected than the non-pedunculated ones [20], [28]. Such a relationship was not confirmed in our study, probably because most of the pedunculated lesions occurring in the study participants were small-sized and had low-grade dysplasia. We also failed to confirm the previously reported associations between a higher likelihood of false-negative FDG PET/CT findings and increased serum glucose levels or obesity [29], [30].

In this study, we identified a total of 10 false-positive FDG PET/CT results. These results are in line with a previous study showing that CRC screening by FDG PET/CT may result in a high rate of false-positive findings because of the uneven physiological FDG uptake, benign adenomas, inflammatory lesions, hemorrhoids, or hyperplastic polyps [16]. In the available literature, there is conflicting evidence on the potential clinical value of the primary lesion SUVmax for differentiating among normal tissue, polyps, and malignant lesions [25], [28], [31]. The results from this study indicate that the SUVmax calculated from FDG PET/CT was lower in participants with false-positive than in those with true-positive findings; however, this difference failed to reach the statistical significance threshold. In contrast, SUVmax values were significantly higher in subjects with adenocarcinomas than in those with non-malignant advanced neoplasms. However, these results should be taken with caution due to the small sample size (n = 3). In agreement with previous studies [25], [32], we recommend colonoscopy for further confirmation in subjects with positive results on FDG PET/CT scans.

Double-contrast barium enema (DCBE) and computed tomographic colonography (CTC) are the recommended imaging modalities for the screening of CRCs for asymptomatic adults in North America, but not in Asia [3], [33]. The reported sensitivity of DCBE for the detection of CRCs and advanced colorectal neoplasms is 90% and 50% respectively [34]. Notably, CTC has a better sensitivity (93%) and specificity (97%) for the identification of lesions >1 cm in size [35]. Although FDG PET/CT screening does not require an uncomfortable bowel preparation, it is clear that the sensitivity of this molecular imaging modality is lower than that of DCBE and CTC, especially for lesions ≤1.5 cm in size on colonoscopy.

Our study has several limitations. First, colonoscopy findings were taken as the gold standard. However, the adenoma detection rate on colonoscopy depends on endoscopist-, instrument-, and patient-related factors [36]. Second, long-term follow-up data were not available for all of the participants with false-positive FDG PET/CT findings. Third, we cannot exclude that some lesions have been missed even in subjects with false-positive FDG PET/CT findings. Fourth, ten subjects refused to receive biopsy or polypectomy, which can result in a potential bias. Finally, the number of advanced neoplasms and the frequency of positive FDG PET/CT results were relatively small. This may at least in part explain why we were unable to replicate some previous findings related to the predictors of positive FDG PET/CT results in the screening setting.

### Conclusions

We conclude from the present data that FDG PET/CT screening of advanced colorectal neoplasms is unwarranted, especially in the presence of lesions with an endoscopic size ≤1.5 cm or low-grade dysplasia. Because of the lower detection rates and the higher costs, it is clear that FDG PET/CT is inferior to both DCBE and CTC for the screening of advanced colorectal neoplasms.

## Supporting Information

Table S1 Characteristics of the study participants with true-positive, false-positive, and false-negative FDG PET/CT results.
(DOCX)
