# Supplementary material for: A Comparison of Positron Emission Tomography and Colonoscopy for the Detection of Advanced Colorectal Neoplasms in Subjects Undergoing a Health Check-Up
Source: PLoS One. 2013 Jul 19;8(7):e69111. doi: 10.1371/journal.pone.0069111 (PMC3716810; doi:10.1371/journal.pone.0069111)
Supplement: Table S1 — Characteristics of the study participants with true-positive, false-positive, and false-negative FDG PET/CT results. (DOCX) [file pone.0069111.s001.docx]

**Table S1.** Characteristics of the study participants with true-positive, false-positive, and false-negative FDG PET/CT results

| **True-positive PET findings** | **Colonoscopy results** | | | **Pathological results** | | **PET results** | |
| --- | --- | --- | --- | --- | --- | --- | --- |
| No. | **Size (cm)** | **Morphology**^a^ | **Location^b^** | **Histological type** | **Degree of dysplasia** | **Location^b^** | **SUVmax^c^** |
| 1 | 1.5 | 0-Ip | D | - | - | D | 5.1 |
| 2 | 4.0 | 0-Is+0-IIa | T | Tubulovillous | High | T | 6.7 |
| 3 | 2.5 | 0-Is | A | Villous | High | A | 7.9 |
| 4 | 4.0 | Ulcerated without definite margins | S | - | Adenocarcinoma | S | 9.2 |
| 5 | 4.0 | Ulcerated with demarcated borders | A | - | Adenocarcinoma | A | 25.0 |
| 6 | 4.0 | Ulcerated without definite margins | S | - | Adenocarcinoma | S | 26.9 |
| **False-positive PET findings** | **Colonoscopy results** | | | **Pathological results** | | **PET results** | |
| No. | **Size (cm)** | **Morphology** | **Location** | **Histology type** | **Degree of dysplasia** | **Location** | **SUVmax** |
| 1 | 0.5 | 0-Is | R | Tubular | Low | R | 5.1 |
| 2 | 0.5 | 0-Is | T | Tubular | Low | T | 5.4 |
| 3 |  | Ulcers | S | - | - | S | 5.1 |
| 4 |  | Diverticulum | A | - | - | A | 7.7 |
| 5 |  | Diverticulum | A | - | - | A | 12.0 |
| 6 |  | Negative | - | - | - | D | 2.6 |
| 7 |  | Negative | - | - | - | A | 5.5 |
| 8 |  | Negative | - | - | - | S | 5.6 |
| 9 |  | Negative | - | - | - | A | 6.2 |
| 10 |  | Negative | - | - | - | A | 7.3 |
| **False-negative PET findings** | **Colonoscopy results** | | | **Pathological results** | | **PET results** | |
| No. | **Size (cm)** | **Morphology** | **Location** | **Histology type** | **Degree of dysplasia** | **Location** | **SUVmax** |
| 1 | 0.5 | 0-Is | A | Tubulovillous | Low | - | - |
| 2 | 0.5 | 0-IIa | S | Tubulovillous | Low | - | - |
| 3 | 0.6 | 0-Is | T | Tubulovillous | Low | - | - |
| 4 | 0.6 | 0-Ip | R | Villous | Low | - | - |
| 5 | 0.7 | 0-Is | R | Villous | Low | - | - |
| 6 | 0.8 | 0-Is | S | Tubulovillous | Low | - | - |
| 7 | 0.8 | 0-Is | S | Tubulovillous | Low | - | - |
| 8 | 0.8 | 0-Is | S | Villous | Low | - | - |
| 9 | 0.8 | 0-Ip | R | Tubulovillous | Low | - | - |
| 10 | 1.0 | 0-Is | C | Tubular | Low | - | - |
| 11 | 1.0 | 0-Is | A | Tubular | Low | - | - |
| 12 | 1.0 | 0-Is | A | Tubular | Low | - | - |
| 13 | 1.0 | 0-Is | A | Villous | Low | - | - |
| 14 | 1.0 | 0-Is | D | Tubular | Low | - | - |
| 15 | 1.0 | 0-Is | S | Villous | Low | - | - |
| 16 | 1.0 | 0-Is | T | Tubular | Low | - | - |
| 17 | 1.0 | 0-Is | D | Tubular | Low | - | - |
| 18 | 1.0 | 0-Is | T | Tubular | Low | - | - |
| 19 | 1.2 | 0-Is | R | Villous | Low | - | - |
| 20 | 1.2 | 0-Is | R | Tubular | Low | - | - |
| 21 | 1.2 | 0-IIa | T | Tubular | Low | - | - |
| 22 | 1.2 | 0-Ip | S | Tubulovillous | Low | - | - |
| 23 | 1.2 | 0-Is | S | - | - | - | - |
| 24 | 1.2 | 0-Ip | T | - | - | - | - |
| 25 | 1.5 | 0-Is | S | Tubulovillous | Low | - | - |
| 26 | 1.5 | 0-Is | C | Villous | Low | - | - |
| 27 | 1.5 | 0-Is+0-IIa | R | Tubular | Low | - | - |
| 28 | 1.5 | 0-Is+0-IIa | R | Villous | Low | - | - |
| 29 | 1.5 | 0-Is | A | - | - | - | - |
| 30 | 1.5 | Polypoid | S | - | Adenocarcinoma | - | - |
| 31 | 2.0 | 0-IIa | T | Tubular | Low | - | - |
| 32 | 3.0 | Ulcerated without definite margins | R | - | Adenocarcinoma | - | - |

^a^Results expressed according to the Paris classification.

^b^C, cecum; A, ascending colon; T, transverse colon; D, descending colon; S, sigmoid colon; R, rectum.

^c^SUVmax, maximal standardized uptake value.
